# Supplementary material for: Predictive data-driven modeling of C-terminal tyrosine function in the EGFR signaling network
Source: Life Sci Alliance. 2023 May 11;6(8):e202201466. doi: 10.26508/lsa.202201466 (PMC10176108; doi:10.26508/lsa.202201466)
Supplement: Supplementary file 3 [file LSA-2022-01466_TableS3.pdf]

A

	**Proliferation**		**Migration**		**Endocytosis**	
	**R2**	**Q2**	**R2**	**Q2**	**R2**	**Q2**
30/30s	0.9427	0.7481	0.9741	0.8204	0.9769	0.8615
60/1m	0.899	0.835	0.9607	0.813	0.978	0.8415
120/2m	0.9443	0.8306	0.986	0.8239	0.9871	0.842
300/5m	0.9606	0.7947	0.9899	0.8607	0.9761	0.7896
Combined	0.9728	0.6547	0.9969	0.811	0.9931	0.7817
AUC	0.9962	0.7326	0.9934	0.8114	0.9945	0.6677

B

	**Proliferation**		**Migration**		**Endocytosis**	
	**R2**	**Q2**	**R2**	**Q2**	**R2**	**Q2**
Combined	0.9728	0.6547	0.9969	0.811	0.9931	0.7817
AUC	0.9962	0.7326	0.9934	0.8114	0.9945	0.6677
